# Factors influencing rheumatologists’ prescription of biological treatment in rheumatoid arthritis: an interview study

**DOI:** 10.1186/s13012-014-0153-5

**Published:** 2014-10-11

**Authors:** Almina Kalkan, Kerstin Roback, Eva Hallert, Per Carlsson

**Affiliations:** Center for Medical Technology Assessment, Department of Medical and Health Sciences, Linköping University, SE-581 83 Linköping, Sweden; Department of Cardiovascular Diseases and Speciality Medicine, University Hospital, SE-581 85 Linköping, Sweden

**Keywords:** Prescription, Rheumatoid arthritis, Biological drugs, Implementation, Physicians, Clinical decision-making, Practice variations, Qualitative, CFIR

## Abstract

**Background:**

The introduction of biological drugs involved a fundamental change in the treatment of rheumatoid arthritis (RA). The extent to which biological drugs are prescribed to RA patients in different regions in Sweden varies greatly. Previous research has indicated that differences in health care practice at the regional level might obscure differences at the individual level. The objective of this study is to explore what influences individual rheumatologists’ decisions when prescribing biological drugs.

**Method:**

Semi-structured interviews, utilizing closed- and open-ended questions, were conducted with senior rheumatologists, selected through a mix of random and purposive sampling. The interview questions consisted of two parts, with a “parallel mixed method” approach. In the first and main part, open-ended exploratory questions were posed about factors influencing prescription. In the second part, the rheumatologists were asked to rate predefined factors that might influence their prescription decisions. The Consolidated Framework for Implementation Research (CFIR) was used as a conceptual framework for data collection and analysis.

**Results:**

Twenty-six rheumatologists were interviewed. A constellation of various factors and their interaction influenced rheumatologists’ prescribing decisions, including the individual rheumatologist’s experiences and perceptions of the evidence, the structure of the department including responsibility for costs, peer pressure, political and administrative influences, and participation in clinical trials. The patient as an actor emerged as an important factor. Hence, factors both at organizational and individual levels influenced the prescribing of biological drugs. The factors should not be seen as individual influences but were described as influencing prescription in an interactive, nonlinear way.

**Conclusions:**

Potential factors explaining differences in prescription practice are experience and perception of the evidence on the individual level and the structure of the department and participation in clinical trials on the organizational level. The influence of patient attitudes and preferences and interpretation of scientific evidence seemed to be somewhat contradictory in the qualitative responses as compared to the quantitative rating, and this needs further exploration. An implication of the present study is that in addition to scientific knowledge, attempts to influence prescription behavior need to be multifactorial and account for interactions of factors between different actors.

**Electronic supplementary material:**

The online version of this article (doi:10.1186/s13012-014-0153-5) contains supplementary material, which is available to authorized users.

## Background

Innovations are necessary to achieve improved health care. However, the effect, cost-effectiveness, and long-term consequences of new drugs, devices, and procedures are seldom established at time point of introduction. One example is biologic drugs for treatment of rheumatoid arthritis (RA). They were introduced around 2000 and were seen as a major breakthrough. A number of randomized controlled trials showed that biologics reduced disease activity and improved quality of life [1-3]. However, recent research indicates that the effect on RA progression in real life might not be as positive as previously reported in clinical trials [4,5]. Some studies even report outcomes in patients treated with biologics as similar to those achieved in patients treated with traditional disease-modifying antirheumatic drugs (DMARDs), but with biologics at a 30- to 40-fold increased cost [6,7].

The use of biologics has increased rapidly over the last decade, and prescription of these drugs has subsequently been extended to patients with less severe disease than previously [8]. Sweden was among the countries that had the highest prescription levels per capita in Europe [9]. At present, there are nine different biologics on the Swedish market, and this pharmaceutical group accounts for the largest sales in the country, at over 2.5 billion SEK at pharmacy purchasing prices in 2012 [10]. The prescription of biologic RA drugs has varied considerably among regions, despite the existence of guidelines from the Swedish rheumatologic professional organization (2004), from the National Board of Health and Welfare (2010), as well as international guidelines [11-16]. Extensive national and regional registries have enabled follow-up of the treatment of RA patients since the introduction of biologics [17,18]. This follow-up shows that twice as many RA patients per capita receive biologics in regions that prescribe the most as compared to regions that prescribe the least [19].

Attempts have been made to explain the regional differences and what influences prescription decisions [20-26]. However, the prescription of biologics has not been widely studied. A recent Swedish interview study investigated factors influencing treatment of breast cancer and treatment of RA and concluded that drug prescriptions are influenced by local clinical practice, established in a network of prescribers making more or less strict interpretations of the current evidence [27]. The study focused on factors influencing prescription from an administrative and political management levels. As other studies have indicated that the identified differences in health-care practice at the regional level might obscure differences at the individual or departmental level [28,29], it is of value to obtain increased knowledge about factors influencing the individual rheumatologist.

The objective of this study is to identify and explore factors influencing the individual rheumatologist’s decision about prescribing biologics. The diffusion of biologics offers a unique opportunity to study prescription decisions, and this study will contribute to the understanding of how such prescription decisions are made.

## Method

This study is part of a three-phase project in which our overall goal is to characterize rheumatologists’ prescription practices in a larger sample. In this phase, using qualitative interviews [30], we focus on “why” rheumatologists are prescribing biologics to differing degrees. We have chosen to study the choice between traditional DMARDs and biologic drugs, disregarding which brands of biologics are used. Ethical approval for the study protocol was obtained from the Regional Ethical Review Board in Linköping (2013/240-31).

### Study sample

The study was conducted at the rheumatology departments of five university hospitals in Sweden. Health care in Sweden is decentralized to 21 regions called county councils (CCs). Most of the CCs have several hospitals, and 7 CCs have university hospitals. The hospitals were chosen in order to cover different parts of Sweden including both southern and northern areas with diverging population densities [31]. Including only university hospitals could possibly diminish the transferability of the results to settings outside of university hospitals. However, in Sweden, approximately 50% of RA patient visits to rheumatologists occur at university hospitals [32]. Since we were interested in individual, rather than regional, variations, these hospitals were chosen in order to guarantee a similar hospital size and setting for the physicians in terms of research exposure and RA patient workload.

Sampling of participants was conducted using a combination of purposive and random sampling techniques. Purposive sampling allowed us to sample a homogeneous sample of participants who would be able to describe their experiences related to the study question [33]. We deliberately chose senior rheumatologists and excluded physicians who were not specialists in rheumatology or lacked experience managing patients with RA. A list of all registered rheumatologists who were members of the Swedish Society for Rheumatology (SRF) was assessed. Lists from the selected university hospitals were extracted, and potential participants were randomly selected. No specific sample size was set *a priori*, and the sampling of new participants continued until redundancy, when themes were recurring.

### Data collection

After agreement by the head of the department at each clinic, the selected participants were contacted by email. Rheumatologists who agreed to participate were emailed an information document. A telephone or in-person interview was scheduled according to the participant’s availability. Comparisons of telephone and face-to-face interview modes have previously been shown to yield no significant differences in the resulting interview transcripts [34].

Semi-structured expert interviews [35] utilizing closed- and open-ended questions were conducted with the rheumatologists from September 2013 to January 2014. Twenty interviews were conducted by telephone, and the remaining six were face-to-face interviews. All interviews were digitally recorded and transcribed verbatim. Informed consent for recording of the interviews was obtained before each interview. The participants were also informed about how the collected data would be analyzed and presented, with particular emphasis on the fact that identification of individual prescribers would not be possible in the final presentation.

### Study design

The interview questions consisted of two parts, using a “parallel mixed method” approach (see Additional file 1). The parallel mixed method comprises collecting and analyzing qualitative and quantitative data simultaneously and comparing or consolidating the results at the interpretation stage. There can be equal focus on the qualitative and quantitative parts, or focus can be on one part, using the other part as a complement [36]. Since the aim of this study was to explore what influences the individual rheumatologist’s decision about prescribing biological drugs, the main focus was on the qualitative part of the method.

In the first and main part of the interview questions, the process of prescription decision-making was explored qualitatively, using a combination of inductive and deductive methodologies. The interview started with explorative questions. A semi-structured interview guide was used to allow for increased flexibility and freedom when exploring the prescribing practices. Each topic in the interview guide was completed with open-ended questions to elicit free responses. It was emphasized that focus was on the informants’ own perceptions and experiences concerning how prescription decisions are made. The informants were asked to freely elaborate on factors that influence prescription decisions and what they saw as barriers and facilitators in using biologics.

To meet the objective of this study of identifying and exploring factors influencing the individual rheumatologist’s decision about prescribing, we used a comprehensive implementation framework, the Consolidated Framework for Implementation Research (CFIR) [37]. Many theories about implementation have been presented, but they differ in definitions and terminologies while also exhibiting extensive overlap. The CFIR was established in order to comprise common constructs found in published implementation theories. The CFIR is a pragmatic “meta-theoretical” framework—it includes constructs from a synthesis of 19 theories about dissemination, innovation, organizational change, implementation, knowledge translation, and research uptake. This framework reflects a “professional consensus” in that it specifies a consistent list of constructs that are believed to influence implementation, but it does not specify in what way (positively or negatively) these constructs influence implementation. An alternative approach would have been to choose one implementation theory, but the overarching typology of CFIR fitted our explorative purposes in this study. Since it encompassed a range of concepts, the CFIR supports the exploration of essential factors that may be encountered during implementation. It has previously been used to explore important implementation factors in several disciplines [38-40].

Follow-up questions were derived deductively from the CFIR and prior literature [19,20,37]. The CFIR constructs are organized into five major domains: 1) the characteristics of the innovation (e.g., evidence strength), 2) the outer setting (e.g., peer pressure), 3) the inner setting (e.g., culture), 4) the characteristics of the individuals involved (e.g., knowledge), and 5) the process used to implement the innovation (e.g., engaging opinion leaders).

In the second part of the telephone interview, after the open questions, the informants were asked to verbally rate predefined factors derived from the CFIR model and previous research [27,37,41]. The physicians were asked to what extent they believed that the various factors influenced prescription decisions (not at all, to some extent, quite a lot, to a large extent). By combining qualitative and quantitative data, an overall or negotiated interpretation of factors influencing prescription can be forged [42]. The qualitative approach was used to elicit which factors the rheumatologists believed influenced the prescription decision, while the complementary rating was used to elicit, quantitatively, how large an impact they believed various factors had.

### Pilot

The questionnaire was pretested in three pilot interviews with senior rheumatologists and was thereafter slightly adjusted. Some factors in the rating were deleted as they were perceived as irrelevant by the rheumatologists. For example, the fifth construct in the CFIR model, the process of implementation, was not used in the rating since the test subjects found it difficult to rate. The construct of process was instead explored in the open-ended questions and was brought up as an integrated part in the other constructs. Damschroder et al. point out that the CFIR model should not be applied wholesale to every problem, but rather in the context of the study [37]. After testing and adjustment, the questionnaire was found to be valid for the research questions in the present study.

### Analysis

Transcripts were read through several times for understanding and to establish an initial coding scheme. The transcripts were then organized using the NVivo software and analyzed using qualitative content analysis in accordance with Hsieh and Shannon [43]. Qualitative content analysis is a method for explorative and descriptive analysis of transcripts based on empirical data [44]. The interviews were analyzed by the main author, who consulted the rest of the project group members for alternative interpretations of the data [45]. The coding scheme was developed gradually, clustering the themes that emerged in the data, all while looking for disconfirming data. The clustered themes corresponded to the categories in the CFIR model, which was thereby confirmed as a helpful tool in the analysis [37]. The quantitative findings were analyzed using standard descriptive statistics (mean, min-max, and standard deviation) in order to summarize and illustrate the features of the data. Integration of the qualitative and quantitative findings was done during the final interpretation and analysis of the data, by comparing the themes in the qualitative part with the quantitative rating. Anonymous codes are used when presenting prescribers’ quotations, for example, “P3, CC1” for “physician 3, county council 1”.

## Results

In total, 105 senior rheumatologists were contacted, and 26 rheumatologists (15 women and 11 men) with equal representation from all five university hospitals were interviewed (Table 1). Among those who replied that they could not participate, the main reasons were a lack of time, parental leave, or retirement. Interviews ranged in duration from 26 to 64 min with an average of 37 min. The rheumatologists had, on average, been active as medical doctors for 22 years, and for the majority, RA patients represented more than half of their total number of patients. The vast majority of the rheumatologists had a doctoral degree in medicine, and most of them were still active in research. Nine rheumatologists were involved on a local or national level in developing clinical guidelines for treatment of patients with RA.

**Table 1:** **Participant characteristics**

	***N*** **= 26 (%)**
Gender (female)	15 (58%)
Mean age in years (range)	53 (41–68)
Years in practice	
1–10	3 (12%)
11–20	10 (38%)
21–30	6 (23%)
30+	7 (27%)
Mean proportion of RA patients in total number of patients	54%
Doctoral degree	22 (85%)
Active in research	19 (73%)
Active in RA guidelines development (local or national)	9 (35%)

### Mapping of prescription factors using the CFIR framework

Prescription factors were grouped into categories based on the CFIR [21] and are presented as they were mentioned and rated by the rheumatologists: 1) intervention characteristics, 2) the prescriber, 3) the patient, 4) the inner setting, and 5) the outer setting (Table 2). The construct of “process” did not emerge as a singularly clear and independent construct in this study. Some parts of “process” inevitably came up and are presented as they occurred, integrated in the other constructs. Since the patients emerged as an important factor, they are presented as a separate category in our study.

**Table 2:** **Summary of the factors influencing prescribing by thematic categories**

**Category**	**Theme**	**Quotations**
1. Characteristics of the intervention	Scientific evidence of effect	“There’s been so much data on the … biologics, their effectiveness … and even side-effect profiles and above all perhaps the absence of serious side effects have meant that they have come earlier and earlier in the treatment arsenal.” (P24, CC2)
	Cost of the drug	“The striking fact about the biological drugs when they arrived was that they were very expensive. So this led to far more expensive treatment than before.” (P10, CC3)
2. Characteristics of the individual prescribers	Knowledge and beliefs about biologics	“How much you know about the drug, how much experience you have with it…If you have something that’s gone wrong, you think it might be the same for the next patient…experiences and habit largely affect prescribing.” (P6, CC3)
	Personal attributes	“Some physicians are also very conservative, they use what they’ve learned … and don’t want to test something else.” (P24, CC2)
3. The patient	Patient characteristics	“The patient influences the decision the most, it all starts there…Because then I can always justify my decision if somebody questions it and says that I prescribed something very expensive, I can always say ‘we chose this based on the patient’ and then I can’t get any criticism for it. The patient is the factor with the most influence.” (P3, CC4)
	Patient as an actor in decision- making	“But then of course there’s also some pressure from the patients. They’re very well informed. And if you decide not to prescribe biologics you’ll presumably have to motivate that decision.” (P26, CC2)
4. Inner setting—the department	Structure of the department	“Another reason (for the varying prescription decisions) is that the number of rheumatologists varies substantially across the country, and if you’re not a specialist in rheumatology you probably don’t feel very accustomed to using that type of drug.” (P23, R2)
	Networks and communication	“When we want to start biological medication in a new patient, we have a group discussion and the decision is made in the group… to get a common practice for the patients we treat. The choice of treatment is still made by the individual physician so it’s more about the decision that it’s okay to start with a biological drug for this patient.” (P12, CC1)
	Leadership engagement	“One thing that influenced prescribing was that I was the medical chief [at the rheumatology department] and I sat down and prioritized the extra resources needed for the drugs at our department.” (P23, CC2)
	Available resources	“We have the pharmaceutical budget, we have had a … well, a limited budget, simply. And these treatments are expensive of course and it’s hard to stick to the budget.” (P11, CC3)
	Culture	“When discussing a patient that I want to put on biologics, my colleagues usually question whether I’ve tried this and that first… My opinion is that it’s more restrictive that way.” (P15, CC1)
5. Outer setting	Cost responsibility	“… I think that the budget still plays a role there… the stricter the budget you have at the department, the more careful you are with the money. I think there are other departments with higher prescription levels that do not really … carry their own drug costs in the same way we do and of course that facilitates their prescribing more.” (P5, CC4)
	Political and administrative influence	“Sometimes you’re lucky and this is an area that is highly prioritized by the county council and then you’re more willing to start treatments, but in other regions it has been quite the opposite and starting treatments has been banned, yes, there has simply been a total stop.” (P20, CC5)
	External policies	“When the new guidelines came there was a clear increase in funding for the drugs in the region… then we also had the new guidelines from the SRF and they could of course also be used as an argument that there were many more [patients] that should have biological drugs so then we need to have more money.” (P18, CC5)
	External peer pressure	“And that was actually what made it possible to increase our previous low level of prescription. When the Open Comparisons came they made it a bit freer to prescribe the drugs.” (P10, CC3)
	Participation in clinical trials	“The studies facilitated understanding of the new drugs, because when you test the new drugs in clinical trials, you’re always in the forefront of development, so we were familiar with the new drugs before they were even available. And I think that has facilitated their use.” (P21, CC5)
	Influence from the pharmaceutical industry	“If companies are very active in marketing at one department, then that can have an impact. Or if there are some personal ties between [physicians and reps] and they think they are nice or something. Then you can get a higher prescription level.” (P7, CC3)

#### Intervention characteristics

##### Scientific evidence of effect

Overall, the rheumatologists described a pattern where the decision to use biologics was largely influenced by scientific evidence about the drugs. When biologics were introduced in the market, many rheumatologists were somewhat doubtful about using them.

Gradually, when an increasing number of clinical studies demonstrated good clinical effectiveness as well as acceptable side effects of biologics, prescriptions increased. In subsequent years, patients were prescribed biologics at an earlier stage of the disease than previously (see Table 2 for quotations). A few rheumatologists pointed out that recent studies supported the use of a combination of traditional DMARDs as an alternative to biologics.

Some rheumatologists pointed out that the various treatment strategies have not been compared and that more evidence about effects is needed, as well as comparisons of the different strategies and evaluation of the long-term effects of the different drugs.

##### Cost of the drug

In addition to scientific evidence about the effect, most rheumatologists emphasized the substantially higher costs of biologics compared to treatment with traditional DMARDs. The higher costs made them think twice before prescribing biologics, since they knew that prescription of these drugs would imply a significant budgetary impact.

#### Characteristics of individual prescribers

##### Knowledge and beliefs about biologics

All respondents recognized that the individual rheumatologist’s subjective judgment and experiences of the drug influenced prescription decisions in several ways. A common argument was that if the drug had good effect in one patient and no serious side effects emerged, the physician felt safe in choosing that drug for the next patient.

##### Personal attributes

There were also a number of personal attributes besides the prescriber’s own experience that were mentioned as influencing factors. Several respondents believed that prescribing was influenced by how well informed and up-to-date the rheumatologists were. Some recognized that prescribers’ attitudes towards innovations influenced their prescribing behavior. They argued that risk-prone physicians were more willing to use the new biologics, while more conservative physicians preferred traditional treatment with which they were familiar. A few respondents also mentioned years in practice as an influencing factor and suggested that junior physicians were more focused on using biologics.

#### Patient characteristics

##### Doctor-patient perspective: medical attributes

All respondents emphasized that the decision to prescribe biologics was largely influenced by patient characteristics such as disease activity, joint destruction, laboratory data, comorbidity, possible infections, as well as the patient’s own assessment of the disease, where patients with higher disease activity were prescribed with biological drugs to a greater extent.

##### Doctor-patient perspective including non-medical attributes

The age of the patient was considered important, as well as the patient’s level of education, since that could have a possible impact on compliance with taking the drug. How the medication would affect the patient’s everyday life was also a factor when considering treatment with biological drugs.

##### Doctor-patient perspective and a wider societal perspective

Some physicians reported that when they were making prescription choices, they also took into account whether the patient would be able to return to work or not. They emphasized that if the patient would be able to go back to work, treatment with biologics might be worth the increased cost.

##### The patient as an actor in decision-making

Several physicians mentioned that the patient’s own will and preferences were important factors. Many patients were well informed about biologic drugs. They were very active in the choice of drug and made demands. Hence, the patients influenced the prescription decisions to a large extent, emphasizing their clinical indications as well as their own preferences.

#### The inner setting

##### Structure of the department

The number of rheumatologists working at the department was emphasized as an influential factor. Departments with a larger number of rheumatologists were considered to be more familiar with biologics and would therefore prescribe them to a larger extent. Further, departments with many rheumatologists were said to allow more frequent visits by the patients, making the physicians more inclined to prescribe biologics. By contrast, some rheumatologists believed that working at a smaller department was associated with greater influence from pharmaceutical sales representatives as compared to larger departments where there were more discussions with colleagues.

##### Networks and communication

Regular meetings with colleagues were also reported to influence prescribing behavior. Some departments had regular meetings where patient cases and drug prescription were discussed. In some regions, there were weekly meetings where the rheumatologists had the opportunity to present patients who were possible candidates for treatment with biologics. Different treatment strategies were discussed, and the various rheumatologists could express their opinions on treatment choice. The physicians contended that such meetings contributed to a higher degree of consensus on prescription strategies.

Informal discussions with colleagues were also said to influence prescription. The physicians pointed out that they shared information and knowledge within their professional network on a regular and informal basis. The influence of senior physicians was particularly prominent, and many physicians emphasized that they learned from more experienced colleagues.

##### Leadership engagement

The rheumatologists reported that the attitude of department management had significant influence and could either facilitate or inhibit prescription of biologics. Some chief managers promoted biologics externally to obtain increased funding from CC management, and they could also allocate resources in the department specifically for biologics.

In other CCs, department directors had limited interest in allocating funds for biologics. If prescription of biologics increased, the physicians were questioned, which discouraged them from prescribing the drugs.

##### Available resources

Many rheumatologists had experienced that staffing and financial resources at the department influenced the extent to which they prescribed biologics. Some physicians expressed experiencing restrictions and that there was a general expectation that decisions about prescribing biologics should be considered carefully.

Physicians gave examples of the budget in some CCs covering only short periods of time. In those cases, they had to discontinue a prescription for one patient in order to be able to start a new patient on biologics. Some argued that the economic restrictions encouraged physicians to consider their decisions carefully.

##### Culture

Some rheumatologists also maintained that there were different local treatment traditions and different opinions about how early in the disease course biologics should be instituted. At some departments, the rheumatologists had a tradition of first trying traditional DMARD therapy, with the possibility of changing one DMARD to another if the first one did not work. If this was not successful, two or three DMARDs could be used in different combinations. If disease activity still persisted, treatment could then proceed to biologics. Other departments, however, initiated biologics after trying only one single DMARD.

Some rheumatologists pointed out that university hospitals had created a culture of early adoption of innovative treatments in general. Therefore, they tested drugs, also including other treatment indications, earlier than what might be done elsewhere.

#### The outer setting

##### Cost responsibility

One common argument among the rheumatologists was that much of the variation in prescribing was caused by differing reimbursement regimens for biological drugs. Previously, there were three types of regimens. CC5 included costs for biologics within a global budget for drugs; CC2 covered outpatient costs within a global budget, while inpatient costs were attributed to the rheumatology department; and in CCs 1, 3, and 4, the rheumatology departments were entirely responsible for the costs. At the time point for data collection, cost responsibility lays with the rheumatology departments in all CCs, apart from CC2 where 50% of the outpatient costs were within the global budget. Many physicians argued that responsibility for the drug budget at the department level had lowered the prescription levels.

##### Political and administrative influence

Besides principles for reimbursement, the rheumatologists described that different forms of management at the central political and administrative levels of the CCs influenced prescription. Firstly, the rheumatologists emphasized that prescribing was influenced by how rheumatology was prioritized compared with other clinical areas in the CCs.

Secondly, in some CCs, there had initially been a liberal attitude towards using biologics, resulting in extensive prescription of these drugs compared to other CCs. After the gradual increase in prescriptions and costs, the CC management imposed restrictions on prescribing biologics.

Thirdly, examples were also given of CC management increasing reimbursement for the rheumatology department after comparisons with treatment practices in other CCs.

##### External policies

The respondents reported trying to follow the guidelines from their professional organization, SRF, as much as possible. The guidelines were often discussed within the medical group, and in some CCs, the respondents claimed that all rheumatologists at the department annually scrutinized the guideline recommendations and updates. According to some physicians, the national guidelines from the National Board of Health and Welfare, published in 2010, have resulted in certain changes in local policy and have influenced prescription by being used as an argument to increase the funding for the department.

##### External peer pressure

Several rheumatologists mentioned the influence of “Open Comparisons” on prescription levels. “Open Comparisons” is an annual report from the Swedish Association of Local Authorities and Regions that compares Swedish CCs with respect to quality of medical care, accessibility, results, and costs for various diseases. The first report concerning biologic drugs came in 2008 and revealed large differences between CCs in prescription of these drugs. In CCs that lagged behind, the political message was raised that all residents are entitled to equitable health care and an increased prescription was encouraged.

##### Participation in clinical trials

Participation in studies on biologics was emphasized as an incentive for increased prescription. The hospitals in CCs 2 and 5 were already participating in clinical studies on biologics in the 1990s. This meant that rheumatologists in these CCs had been treating several patients with biologics, and they were well prepared to continue with these treatments when the drugs became available on the market.

Clinical registers were created to follow the effects of biologics on patients. As a consequence of the follow-ups, it also became easier to argue for more resources when different departments were compared. They used the results as an argument for themselves, for the patients, and for politicians. Hence, the clinical trials and the consecutive register follow-ups increased the prescription of biologics.

##### Influence from the pharmaceutical industry

Some respondents also believed that there was external influence from pharmaceutical representatives through their promotional activities directed toward physicians and rheumatology departments. There were examples in all CCs of sales representatives organizing information meetings and lunches to promote the drugs and to disseminate the latest results from their trials. The companies also participated at several conferences with training sessions and general sponsoring.

### Rating of predefined factors that influence prescribing practice

In the respondents’ rating of predefined factors that may influence the prescription decision, three broad categories of factors can be distinguished (Table 3).

**Table 3:** **Rating of factors that influence prescription decisions**

**Factor**	**Mean rating**	**Min-max**	**SD**
Proven effect and patient benefit of the drug	2.96	2–3	0.19
Strength and quality of the scientific evidence	2.85	2–3	0.36
Prescriber’s knowledge and experience	2.52	1–3	0.58
Patient’s level of disease activity	2.40	0–3	1.01
National and professional guidelines	2.39	1–3	0.63
Colleagues	2.11	1–3	0.58
Local guidelines	2.08	0–3	0.92
Prescriber’s attitude to biologics	1.96	0–3	0.98
Cost-effectiveness of biologics	1.93	0–3	0.78
Formal leaders at the department	1.85	1–3	0.66
Economic resources and pharmaceutical budget	1.78	0–3	0.75
Cost of the drug	1.70	0–3	0.87
Economic consequences for the department	1.70	0–3	0.87
Study participation by the prescriber	1.44	0–3	0.88
Informal leaders at the department	1.41	0–3	0.84
Feedback (from colleagues, leaders, statistics)	1.31	0–3	0.61
Mode of prescription (if taking it is complicated)	1.29	0–3	0.70
Patient’s expressed requests and wishes	1.04	0–2	0.50
Non-disease-related attributes of the patient	0.93	0–3	0.62
Information/marketing from the pharmaceutical company	0.81	0–2	0.48
Media attention in newspapers, TV	0.43	0–1	0.49

Influences to a large extent = 3, quite a lot = 2, to some extent = 1, not at all = 0. *N* = 26.

Among the factors rated by the rheumatologists as having the highest impact on the prescribing decision, are those concerning scientific evidence and professional and national guidelines. More precisely, they concern the evidence of the efficacy and patient benefit of the drug, the strength and quality of the scientific evidence, the prescriber’s knowledge and experience, the patient’s level of disease activity, the influence of colleagues, and national, local, and professional guidelines.

Factors rated as having moderate influence are associated with formal and informal influences from leaders at the department, cost and budget issues, and the prescriber’s own attitude and experiences from participating in studies of biologic drugs.

Finally, factors rated as having the lowest impact on prescription decisions are the patients’ expressed requests and wishes and non-disease-specific patient attributes, as well as influence from pharmaceutical companies and media.

## Discussion

The present study presents a cascade of factors, besides the clinical indications, that influence prescription of biologics. From the perspective of Swedish rheumatologists, these factors comprised characteristics of the drugs; the actors involved, including the patients; the inner setting; and the outer setting. Thus, the factors could be categorized in the Consolidated Framework for Implementation Research [37]. Given the broad range of factors that were mentioned as influencing the prescribing of biologics, the large regional variations are not unexpected. Since these factors differ between CCs, departments, and prescribers, they contribute to varying levels of prescription.

The scientific evidence on the effect of the drugs was both described and rated as having a highly significant influence on prescription. However, general agreement on the evidence for biologics was lacking, and there were diverging interpretations. Some physicians pointed out that research supports the use of biologics, while, on the contrary, others pointed out that several recent studies have demonstrated similar outcomes using combination treatment with traditional DMARDs. Different physicians emphasized different aspects of the evidence base to strengthen their argumentation. The diverging views might reflect that some applications of biologics are still relatively new, with growing evidence leading to the question if the initial general expectations of effect and cost-effectiveness of biologics are being fulfilled.

In addition, the diverging views further emphasize that other issues, besides evidence and guidelines, are of importance in clinical decision-making. Subjective judgments and experience were emphasized by the rheumatologists, and the factor “prescriber’s knowledge and experience” was the third highest rated in the quantitative rating. According to Prosser et al. [23] and Prosser and Walley [24], four types of knowledge are important influences on new drug uptake: scientific, social, patient, and experiential knowledge. Although scientific evidence is cited as the key source for new drug uptake, it is combined with the other forms of knowledge. Gaps in scientific knowledge can be filled through professional networks (“social knowledge”) and previous experience (“experiential knowledge”), which can give rise to the variations seen in clinical practice. This was certainly the case when biologics were introduced in the market but is still of importance, leading to variations between regions as well as between individual physicians. Patient knowledge was also important in the present study and will be discussed in more detail later.

The importance of social knowledge, such as professional networks and collegial discussions, was raised by many physicians. Colleagues were also quantitatively rated as having a relatively high influence on the prescription decisions, right after scientific evidence, prescriber’s knowledge and experience, patient’s level of disease activity, and national and professional guidelines. The physicians often discussed alternative strategies with colleagues before making a decision. The importance of colleagues has also been shown by others [25,46]. McGettigan et al. have suggested that “the medium is more important than the message” [47]. In a survey comprising 230 hospital physicians, the vast majority declared that their primary sources of information on new drugs were colleagues and clinical meetings [47]. This may contribute to therapy traditions being preserved within the clinic and indicates the importance of the characteristics of the inner setting.

Several factors at the organizational level were also mentioned as influencing prescription decisions. The vast majority of the rheumatologists mentioned the impact of available resources for drugs, as well as how the costs were allocated, as influential factors. Cost-effectiveness of biologics, economic resources and pharmaceutical budget, cost of the drug, and economic consequences for the department were all factors that were rated as having medium influence on the prescription decision, higher than, e.g., informal leaders at the department. In previous research, financial incentives such as budget responsibility have also been mentioned as having an influence on the uptake of new drugs [41]. A recent Cochrane review showed that the mere presence of financial incentives may influence prescriptions [48]. Based on 13 studies, this review concluded that when a group of or individual physicians manage their own budget, they prescribe fewer and less expensive drugs. Our study covered both departments that had experience of having costs for biologics included in a global budget at the region, and those having drug cost responsibility at the rheumatology department. Many physicians argued that responsibility for the drug budget at the department had lowered the prescription levels.

Treatment guidelines were rated of similar importance as the patient’s level of disease activity. National registries and audits through the Open Comparisons Reports were also mentioned as a powerful factor and a tool for making administrative leaders and policy makers aware of variations in prescriptions, which in turn led to increased resources for biologics in CCs that lagged behind.

Participation in clinical trials with biologics influenced prescription both at an individual and an organizational level but was rated as having a quite low influence. However, respondents described in interviews how the physicians’ awareness and experience of the drugs made them more prone to use them after participating in trials. Corrigan and Glass showed that clinical trial investigators often act as “early adopters”, and their use can affect the behavior of other physicians [49]. Damschroder et al. also include “intervention source”, defined as the perception among potential users of whether the innovation is externally or internally developed, as an innovation characteristic that might influence adoption [37]. Participating in clinical trials probably increased the perception among rheumatologists that the biologics were developed internally, and this strengthened the legitimacy of the drugs for further use. At a CC level, results from trials were used as an argument for increased funding for the rheumatology departments. Furthermore, use of biologics was facilitated by sponsoring of the drugs and other promotional activities by the pharmaceutical companies. This may be in contradiction to the low rating of influence from the pharmaceutical companies.

A striking finding in the present study is that patient attitudes and preferences were considered to influence the prescription decision by many respondents, although patient preferences were rated relatively low. Our finding might reflect two conflicting mechanisms. On the one hand, they might reflect the changes over time in the attitudes of clinicians concerning the appropriateness of involving patients in the decision-making. Patient involvement is today largely discussed, which could explain why it is also mentioned to be influential in the interviews. On the other hand, there is a simultaneous discussion about fair treatment and the physician’s autonomy towards the influence of individual patients. This might explain the relatively low rating of patient preferences. In most studies, patients are mentioned merely as one of the many actors influencing the decision regarding the use of new medicines [41]. The present study deals with RA, which is a chronic disease. Long relationships are often established between patient and physician, and this may contribute to patient involvement being in focus. This has also been mentioned in a recent study of factors influencing prescribing for early RA, where rheumatologists indicated that demographic (e.g., older patients), socioeconomic (e.g., patients with lower income), and psychosocial characteristics (e.g., anxious patients) were factors that were taken into account in the final decision [26].

Certain aspects of the CFIR model that were expected to have an influence on prescription, e.g., complexity of the intervention, implementation climate, and process, were excluded after the pilot study. Following Damschroder et al. [37], we considered it important to apply the model in the context of the study. Other factors, e.g., feedback, the patient’s expressed requests, and information from the pharmaceutical company, were rated unexpectedly low by the rheumatologists, especially since they were repeatedly mentioned in the open interviews.

The qualitative approach of the present study is both a strength and a drawback. It works well in fulfilling the main purpose of increasing the understanding of possible factors which could influence prescribing practice. However, it might not reveal actual behavior. There is a potential risk of response bias, with the respondent not being aware of, or even wanting to admit, what influences his/her behavior. Accordingly, the responses in the interview and the rating may be biased, and there might be an effect of social desirability at play. We cannot rule out that we have missed important factors in the analysis of the data, despite carefully reading the original transcripts several times after categorizing the themes. The transferability of this study to settings outside of university hospitals might have limitations. However, in Sweden, approximately 50% of RA patient visits to rheumatologists occur at university hospitals [32]. Future research about factors influencing prescription decisions could also consider complementary approaches, such as hypothetical patient cases or registry studies.

The various factors that were identified act, and have their influence, at either an organizational or an individual level. The organizational factors can be suspected to cause much of the variation between different areas, but individual factors probably strengthen regional variations and cause the variations between physicians at the same department. The factors should not be seen as single influences, but rather as acting in an interactive, nonlinear manner, where one factor could have an immense influence on the others and causes a chain reaction in one setting, while barely making an impact in another setting. The importance of practice setting has been emphasized previously in implementation studies [50,51] but merits further investigation in view of its great significance.

An implication of the present study is that, in addition to scientific knowledge, attempts to influence prescription behavior need to be multifactorial and account for interactions of factors between different actors. Personal knowledge and experience have been demonstrated to play a major part in prescription decisions. This raises concerns about the need for further research to determine the best use of biologics in individual RA patients and a more efficient diffusion and implementation of current knowledge. The existing variation in biologic treatment might imply that some patients are being overtreated, whereas others are being undertreated, none of which might be optimal for the balance between costs and patient benefits. The involvement of patients was mentioned as an important factor. Therefore, more research on development of shared decision-making is called for. Further qualitative and quantitative studies of individual prescription patterns are needed to investigate actual behavior and to assess the importance of various factors influencing prescribing decisions.

## Conclusion

Differing perceptions of the evidence emerged among individual rheumatologists, and different aspects of the evidence base, such as arguments for biologics or for traditional DMARDs, were accentuated in order to strengthen individual stances. These emerged as an important cause of variations in prescribing at the individual level.

The physicians’ experiential knowledge reinforced their prescription patterns, and positive previous experiences increased their willingness to prescribe the drugs. Rheumatologists were also influenced by local clinical practice, established in their own network.

Furthermore, the patients emerged as an important factor. Patient attitudes and preferences seemed to influence many prescribers, although patient preferences were rated relatively low.

At an organizational level, administrative and political managements were influenced by the magnitude of prescriptions in comparable regions. Public attention to the unequal provision of biologics contributed to policies being changed to harmonize prescription levels with those in other regions.

Hence, a constellation of various factors both at individual and organizational levels, and their interaction, influenced rheumatologists’ prescribing decisions. The CFIR model was helpful in understanding the various factors influencing rheumatologists’ prescription practice regarding biologics.

## Additional file

Additional file 1:
**Questionnaire.** This file contains interview questions used in this research.

## Abbreviations

CC: county council
CFIR: Consolidated Framework for Implementation Research
DMARD: disease-modifying antirheumatic drugs
RA: rheumatoid arthritis
SRF: Swedish Society for Rheumatology
