# Supplementary material for: Factors influencing rheumatologists’ prescription of biological treatment in rheumatoid arthritis: an interview study
Source: Implement Sci. 2014 Oct 11;9:153. doi: 10.1186/s13012-014-0153-5 (PMC4200139; doi:10.1186/s13012-014-0153-5)
Supplement: Additional file 1: — Questionnaire. This file contains interview questions used in this research. [file 13012_2014_153_MOESM1_ESM.docx]

**SUPPLEMENTARY MATERIAL 1- QUESTIONNAIRE**

- Hello, my name is [name of interviewer]. *Introduce oneself*
- *Introduce the study* (Participants were emailed an information document before the interview).

This study is part of my PhD project about implementation in health care. The specific focus of this study is regional variation in drug prescription and the aim is to explore the variation in prescription of biological drugs for RA. The study is managed independently as part of my PhD thesis and does not have any connections to the pharmaceutical industry or any interest organization.

- *Explain the purpose of the interview*

I would like to use this time to talk to you about prescription, what you see as influential factors on prescription and what you take into account when you prescribe drugs to patients with RA. We have chosen to study the choice between traditional disease-modifying antirheumatic drugs (DMARDs) and biologics, disregarding which brands of biologics are used.

We will be interviewing several physicians at your department to gain multiple perspectives. We will also interview a number of physicians at four other university hospitals. Now we are really interested in learning more about your own experience with prescription.

- *Describe the audio recording and how we will assure confidentiality and answer any questions*

I will start the interview with some general questions about your experience of prescription to RA-patients. Then we will continue with open ended questions about prescription and finally we will talk about specific parts more in detail.

This interview will be audio recorded so that we have an accurate record of your thoughts. Nobody outside of the research team will have access to any of your responses. In any use of your quotations, they will be unidentified and it will not be possible to link the responses to you personally. Our study has an approval from the Regional Ethical Review Board in Linköping. Is it OK that I record the interview?

You may skip any questions you wish during the interview. The interview usually takes about 30-45 minutes.

Do you have any questions for me before we begin? *Answer any questions*

**INTRODUCTION**

- Will you please describe your background and your role within your department?
  - How long have you been active as a rheumatologist?
  - Are you engaged in research?
  - Are you involved on a local or national level in developing clinical guidelines for treatment of patients with RA?
- What proportion of your total number of patients are patients with RA?
- Where do you gain new knowledge about treatment of RA? (Scientific papers, conferences, colleagues, guidelines, pharmaceutical companies?)

**DESCRIPTION OF PRESCRIBING**

- How did your department start to prescribe biologics? How were they introduced?
- How are prescription decisions taken today? How have they been taken previously?
- What influences prescription decisions? What factors do you see are important when prescribing biologics to RA-patients?
  - What influencing factors do you see that are connected to the drug itself?
  - What influencing factors do you see that are connected to the prescriber?
  - What influencing factors do you see that are connected to the department?
  - What influencing factors do you see that are connected to the county council?
  - What factors do you see have facilitated / hindered prescription?
- Do you have any systematic meetings where you discuss the prescription? Have you had such meetings previously? How do you believe they have influenced the prescription?
- Do you see anything that has been controversial with the biologics?
- There are reported variations in prescription of biologics to RA-patients between different regions in the country (Open Comparisons 2012). What do you think can cause such variations?
- Do you think that there are variations **within** regions/county councils? Do you think that there are variations between different departments? Do you think that there are variations between different prescribers? What do you think can cause such variations?

**RANKING OF PREDEFINED FACTORS**

In previous research a number of factors have been identified that could influence prescription, and we would like you to rank the following factors according to how relevant they are in you prescription decisions. We have categorized the factors in characteristics of the drug, the patient, the prescriber, the department and external influence. We would like you to rank whether they influence your prescription to a large extent=3, quite a lot=2, to some extent=1, not at all=0.

| **How are prescription decisions influenced by characteristics of the drug:** | Not at all | To some extent | Quite a lot | To a large extent |
| --- | --- | --- | --- | --- |
| Proven effect and patient benefit of the drug |  |  |  |  |
| Strength and quality of the scientific evidence |  |  |  |  |
| Cost-effectiveness of biologics |  |  |  |  |
| Cost of the drug |  |  |  |  |
| Mode of prescription (if taking it is complicated) |  |  |  |  |
| **How are prescription decisions influenced by the patients** |  | | |  |
| Patient's level of disease activity |  |  |  |  |
| Patient's expressed requests and wishes |  |  |  |  |
| Non-disease related attributes of the patient |  |  |  |  |
| **How are prescription decisions influenced by the prescribers** |  | | | |
| Prescriber's knowledge and experience |  |  |  |  |
| Prescriber's attitude to biologics |  |  |  |  |
| Study participation by the prescriber |  |  |  |  |
| **How are prescription decisions influenced by the the department** |  |  |  |  |
| Colleagues |  |  |  |  |
| Formal leaders at the department |  |  |  |  |
| Informal leaders at the department |  |  |  |  |
| Economic resources and pharmaceutical budget |  |  |  |  |
| Economic consequences for the department |  |  |  |  |
| Feedback (from colleagues, leaders, statistics) |  |  |  |  |
| **How are prescription decisions influenced by external factors** |  | | |  |
| National and professional guidelines |  |  |  |  |
| Local guidelines |  |  |  |  |
| Information/ marketing from the pharmaceutical company |  |  |  |  |
| Media attention in newspapers, TV |  |  |  |  |
| **Which other factors could influence prescription?**  **Which three factors influence the most?** |  |  |  |  |
